# Epidemiology of cerebral palsy among children in Ghana

**DOI:** 10.4102/ajod.v13i0.1336

**Published:** 2024-09-20

**Authors:** Israt Jahan, Sk. Md. Kamrul Bashar, Francis Laryea, Samuel Kofi Amponsah, Frederick Inkum Danquah, Mohammad Muhit, Hayley Smithers-Sheedy, Sarah McIntyre, Nadia Badawi, Gulam Khandaker

**Affiliations:** 1 Child Sight Foundation, Dhaka, Bangladesh; 2 Asian Institute of Disability and Development (AIDD), University of South Asia, Dhaka, Bangladesh; 3 School of Health, Medical and Applied Sciences, Central Queensland University, Rockhampton, Queensland, Australia; 4 Korlebu Teaching Hospital, Accra, Ghana; 5 Department of Health Information, Christian Health Association of Ghana, Accra, Ghana; 6 St. John of God College of Health, Duayaw Nkwanta, Ghana; 7 Cerebral Palsy Alliance Research Institute, Specialty of Child and Adolescent Health, Sydney Medical School, Faculty of Medicine and Health, The University of Sydney, Camperdown, New South Wales, Australia; 8 Grace Centre for Newborn Intensive Care, Sydney Children’s Hospital Network, Westmead, New South Wales, Australia; 9 Discipline of Child and Adolescent Health, Sydney Medical School, The University of Sydney, Camperdown, New South Wales, Australia; 10 Central Queensland Public Health Unit, Central Queensland Hospital and Health Service, Queensland, Australia

**Keywords:** disability, cerebral palsy, register, epidemiology, children, low-and middle-income countries

## Abstract

**Background:**

The epidemiology of cerebral palsy (CP) is poorly described in Ghana. These data are crucial for evidence-based intervention for children with CP in the country.

**Objectives:**

We aimed to describe the epidemiology of CP among children in Ghana.

**Method:**

We established the first institution-based register of children with CP in Ghana (Ghana CP Register–GCPR). Children with confirmed CP aged < 18 years were registered following a detailed neurodevelopmental assessment. Socio-demographics, risk factors, predominant motor type and topography, gross motor function classification system (GMFCS), associated impairments, education and rehabilitation status were documented.

**Results:**

Between October 2018 and February 2020, 455 children were registered (mean [standard deviation {s.d.}] age at assessment: 5.9 [4.1] years). Preterm birth and low birthweight were reported in 52.0% and 21.1% children respectively. Most children (79.6%) had a pre- or perinatally acquired CP and the mean (s.d.) age of CP diagnosis was 22.2 (21.6) months. Overall, 55.9% of children had spastic tri- or quadriplegia, 60.5% had GMFCS level III–V and 70.3% had ≥ 1 associated impairment. However, 20.5% had never received rehabilitation services and 69.6% of school-aged children in the GCPR were not enrolled in schools.

**Conclusion:**

The study findings indicate a high burden of severe motor and associated impairment among children with CP in Ghana which highlights the need for tailored interventions to improve health and well-being of children with CP in the country.

**Contribution:**

The study highlights the need for interventions to improve functional outcome, health and well-being of children with CP in Ghana.

## Introduction

Cerebral palsy (CP) is a group of non-progressive but permanent neurological disorders that affects the movement and posture of an individual (Rosenbaum et al. 2007). The primary causes of CP remain unknown in many cases, and the commonly known risk factors of CP include preterm birth, low birthweight, prolonged labour, meconium aspiration, abnormal foetal position, birth asphyxia, neonatal seizure, respiratory distress syndrome, hypoglycaemia and infection or sepsis among children in high-income countries (HICs) (McIntyre et al. 2013). Limited data from low- and middle-income countries (LMICs) suggest that the estimated prevalence of CP is two to three times higher in LMICs than HICs (McIntyre et al. 2022). For instance, the prevalence of CP was reported to be 3.4 per 1000 live births in Bangladesh (Khandaker et al. 2019) compared to 1.4 per 1000 live births in Australia (Australian Cerebral Palsy Register Group [ACPR] 2019).

In addition to higher prevalence, LMICs experience greater risk factors for CP. For instance, the proportion of preterm birth and low birthweight was found to be 16.4% and 37.6% among children with CP in Bangladesh compared to 47.6% and 37.5% respectively among children with pre- and perinatally acquired CP in Australia (ACPR 2018; Khandaker et al. 2019). Furthermore, a regional difference is observed in terms of the burden of different risk factors of CP in LMICs (Jahan et al. 2021b; Kakooza et al. 2017; Khandaker et al. 2019).

Similar differences are also observed in terms of gross motor function limitations of children with CP in different countries. Latest data suggest that 68.2%, 39.0% and 34.7% children with CP in Bangladesh, Uganda and Australia had gross motor function classification system (GMFCS) level III–V (ACPR 2019; Kakooza et al. 2017; Khandaker et al. 2019). Furthermore, a high burden of associated impairments (such as hearing, speech, vision, intellectual functioning and epilepsy) was also observed among children with CP in both LMICs and HICs (Delacy, Reid & ACPR 2016; Narayan et al. 2023). Unfortunately, in LMICs, most of these children with severe motor function limitations lack need-based rehabilitation services (Al Imam et al. 2021b).

The vast difference in the epidemiology of CP (e.g. risk factors, motor function, clinical characteristics) between HICs and LMICs and even within LMICs makes it difficult to utilise the interventions commonly developed and practised in high-resource settings (Paneth 2021). Understanding the epidemiology of CP in different regions could guide the development of need-based early intervention and rehabilitation support using the limited resources available in LMICs. Although a few available studies have reported a high burden of CP in the African region (Abas, Abdelaziem & Kilany 2017; Donald et al. 2014; Duke et al. 2020; Frank-Briggs & Alikor 2011; Kakooza-Mwesige et al. 2017), there is a dearth of knowledge on the risk factors as well as clinical, motor and rehabilitation features of children with CP in Ghana.

Ghana is an LMIC in Western Africa with 13.3% and 23.4% of the population living below the international and national poverty line, respectively (World Bank 2019). The health services in Ghana are delivered via a mix of public (i.e. Ministry of Health – Ghana Health Services [GHS], teaching hospitals, quasi-government institutions and statutory bodies), private sectors (e.g. private-for-profit, mission-based providers, civil society organisations, other private providers) and traditional healers (traditional medicine providers, alternative medicine and faith healers) (University of Ghana [UoG] 2018). Moreover, preventive and curative health services at the sub-district, district and regional levels are delivered by a wide network of outreach services, health centres and hospitals (UoG 2018). Additionally, community-based health planning and services, traditional birth attendants (TBAs) and traditional healers play a crucial role in providing services at the community and household levels (UoG 2018). Despite a government-funded health insurance scheme (i.e. National Health Insurance Scheme [NHIS]) to finance the health care needs of the population in Ghana, latest reports suggest only 36% of the population was supported by this scheme in 2018 (UoG 2018).

Nonetheless, recent studies illustrate how concerted efforts over the previous decade have resulted in a significant improvement in the practice of institutional delivery, skilled birth attendance and antenatal care for children in Ghana (UoG 2018; Ghana Statistical Service [GSS], GHS and ICF International [ICF] 2015, 2018). Yet, national level surveys in the last few years have identified a high proportion of low birthweight, preterm birth and birth asphyxia (i.e. known risk factors) among infants in Ghana (GSS et al. 2015, 2018). In the absence of systematic data collection, it is difficult to predict or monitor the neurodevelopmental outcomes of these children at high-risk of developing CP. A lack of oversight could also delay diagnosis and scope for early intervention which is crucial to improve the functional outcome, health and well-being of children with CP in Ghana (Jahan et al. 2019; Karim et al. 2019a; King et al. 2022; Power et al. 2019).

Cerebral palsy registers can play a significant role in minimising the evidence gap and generating robust data (e.g. prevalence, trend, and aetiology of CP) with positive impact on early diagnosis, early intervention and functional outcome of children with CP. In the last decade, several CP registers have been established in LMICs with the common goal of improving the knowledge base and increasing provision of evidence-based services for children with CP in low-resource settings. In 2018, with support from the Global LMIC CP register (GLM CPR - a multi-country network of > 12 CP registers in LMICs) (Jahan et al. 2021), a group of professionals in Ghana initiated an institution-based register of children with CP, known as Ghana CP Register (GCPR). This study aimed to describe the epidemiology of CP among children in Ghana using data from the GCPR.

## Methods

The GCPR is an ongoing register of children with CP that collects information on selected variables to generate evidence on the epidemiology of CP in Ghana. In this study, we used preliminary data collected as part of the GCPR.

### Study settings and participants

Study participants are children with CP aged < 18 years registered into the GCPR between 2018 and 2020. The GCPR followed an institution-based surveillance method. The register activities were initially started at one facility that is the Salvation Army Rehabilitation Centre, Begoro, and were gradually expanded to the other three facilities selected purposively. These were St. John of God Hospital, Duayaw Nkwanta; Holy Family Hospital, Techiman, Bono East, and Komfo Anokye Teaching Hospital, Kumasi, Ghana. All these facilities provide services to children with disabilities from all around the country.

### Case definition and ascertainment

The case definition was adopted from the Surveillance of CP in Europe (SCPE) and ACPR (ACPR 2019; Cans 2000; Khandaker et al. 2015, 2019). According to the definition, ‘Cerebral palsy – (i) is an umbrella term for a group of disorders, (ii) is a condition that is permanent but not unchanging, (iii) involves a disorder of movement, posture and motor function, (iv) is beecause of a non-progressive interference, lesion, or abnormality, and (v) the interference, lesion, or abnormality originates in the immature brain’ (Khandaker et al. 2015).

An opportunist recruitment strategy was used to recruit children into the GCPR. All children with a confirmed diagnosis of CP attending the inpatient or outpatient services at the participating healthcare facilities during the study period were eligible to participate.

### Data collection and quality assurance

An assessment team (including a physiotherapist, an occupational therapist, a public health research officer) from the participating centres conducted a detailed neurodevelopmental assessment of each participating child. A standard case record form (CRF) was adapted from the GLM CPR (ACPR 2019; Jahan et al. 2021; Khandaker et al. 2019). Information was collected on socio-demographic characteristics of both children with CP and their families. The known risk factors of CP, predominant motor type, topography and gross motor function, educational and rehabilitation status of the registered children with CP were also documented. The assessors received a 1-day training on the methodology and data collection for the register by an expert trainer. Detailed information about the data-collection technique is given further in the text.

### Sociodemographic characteristics

A trained public health research officer documented socio-demographic characteristics by interviewing the primary caregivers of registered children. Information about parental education and occupation at the time of birth of the child, total household income, total number of household members, housing characteristics and household water and sanitation facilities was collected. The CRF had specific questions adapted from the Ghana Demographic and Health Survey (GDHS) questionnaire to document parental education, household drinking water sources and sanitation facilities (GSS et al. 2015). Drinking water sources such as piped water supply in a dwelling or yard or plot, public tap or standpipe, tube well or borehole, protected dug well, protected spring, rainwater, bottled or sachet water, were categorised as improved sources (GSS et al. 2015), whereas drinking water sources such as unprotected dug wells, unprotected springs and tanker trucks or carts with small tanks, and surface water were categorised as unimproved sources of drinking water (GSS et al. 2015). A toilet facility with flush or pour flush to a piped sewer system or septic tank or pit latrine, ventilated improved pit latrine and pit latrine with slab were documented as improved sanitation facilities (GSS et al. 2015).

### Known risk factors

Information related to the aetiology or risk factors of CP was documented by interviewing the primary caregivers and reviewing available medical records. A standard CRF adapted from the GLM CPR was used to collect information on (1) maternal characteristics (age at birth of the child, consanguinity, place of birth, mode of delivery), (2) clinical characteristics (e.g. gestational age, birth weight, plurality), (3) other pre- or perinatal factors, for example, signs of birth asphyxia, early feeding difficulties, any complication during the child birth and (4) timing of the brain injury among participating children.

*Gestational age* was categorised into four groups as follows: (1) term > 37 weeks, (2) moderate to late preterm (32–37 weeks), (3) very preterm (28–31 weeks), (4) extreme preterm (< 28 weeks). *Birth weight* was categorised into two groups: (1) normal birth weight (≥ 2500 g), and (2) low birth weight (< 2500 g). The *timing of brain injury* was documented as (1) pre or perinatal, or (2) post-neonatal (if the brain injury occurred after 28 days of birth or before 5 years of age).

A public health research officer trained in conducting the interviews and reviewing medical records collected this information.

### Motor characteristics and presence of associated impairments

The predominant motor type and spastic topography of CP were documented following the classification used by ACPR and Bangladesh CP Register (BCPR) (ACPR 2019; Khandaker et al. 2015, 2019). The GMFCS level was assessed following standard guidelines (Rosenbaum et al. 2008). The presence of associated impairments (e.g. hearing, speech, visual, intellectual impairment and epilepsy) was assessed and recorded based on clinical examination, review of available medical records and a detailed history provided by the primary caregivers. Furthermore, the severity of any associated impairment present was documented following the classification and guidelines used in BCPR and ACPR (ACPR 2019; Khandaker et al. 2019). A detailed methodology of the assessments is available in Supplementary Table 1.

**TABLE 1:** Socio-demographic characteristics of children registered into the Ghana CP Register (*N* = 455).

Socio-demographic characteristics-subgroups	*n*	%
**Age group (in years) (*N* = 449)†**		
0–4	225	50.0
5–9	149	33.0
10–14	53	12.0
15 and above	22	5.0
**Sex of the child (*N* = 454)†**		
Male	263	57.9
Female	191	42.1
**Mother’s educational level at birth of the child (*N* = 440)†**		
No education	138	31.0
Primary	136	31.0
Secondary	87	20.0
More than secondary	79	18.0
**Father’s educational level at birth of the child (*N* = 433)†**		
No education	85	20.0
Primary	134	31.0
Secondary	90	21.0
More than secondary	124	28.0
**Mother’s occupation at birth of the child (*N* = 454)†**		
Sales and service	182	40.0
Professional or technical or managerial	72	16.0
Skilled manual (e.g. seamstress, hairdresser, baker, cook or chef and others)	65	14.0
Agriculture	64	14.0
Not involved in any work or unemployed	53	12.0
Others	17	4.0
**Father’s occupation at birth of the child (*N* = 446)†**		
Skilled manual (e.g. electrician, painter, plumber, driver, carpenter and others)	140	31.0
Professional or technical or managerial	99	22.0
Agriculture	98	22.0
Sales and service	68	15.0
Not involved in any work or unemployed	8	2.0
Others	33	7.0
**Type of source (*N* = 444)†**		
Pipe borne outside dwelling	213	48.0
Borehole or pump or tube well	117	26.0
Pipe borne inside dwelling	78	17.0
River or stream	34	8.0
Other sources	2	1.0
**Type of facility used (*N* = 451)†**		
Water closet	152	34.0
Pit latrine	129	29.0
Public toilet	124	27.0
Kumasi ventilated improved pit latrine	45	10.0
Other	1	0.2

IGA, income generating activities; IQR, Interquartile range.

† , Total responses for the specific characteristics-subgroup.

The information was collected by direct assessment of participating children by a physiotherapist and an occupational therapist trained on the standard protocol mentioned earlier in the text.

### Education

If a child was aged ≥ 6 years at the time of assessment, their educational status was documented. The caregivers were asked whether the child (aged ≥ 6 years) was enrolled in any mainstream or special education programme. If the response was yes, then the type of school was documented; however, if the response was no, then the reason for not enrolling the child to any education system was recorded. A trained occupational therapist interviewed the primary caregivers and collected this information.

### Rehabilitation status

Access to rehabilitation services was documented for all children included in the GCPR. Information related to the age of receiving rehabilitation for the first time, the type and place of services received were documented for children who ever received any rehabilitation services. The causes of not accessing or utilising any rehabilitation services were documented for children who never received any rehabilitation. A physiotherapist collected information related to rehabilitation status by interviewing the primary caregivers of participating children.

### Statistical analysis

Descriptive statistics were used to report the socio-demographic characteristics, known risk factors of CP, motor characteristics, rehabilitation and education status of children with CP registered into the GCPR. Missing data for any variable were indicated using footnotes, and valid percentages were reported throughout the results section. All analyses were completed using SPSS version 26 (IBM Corporation, Chicago, IL).

### Ethical considerations

Ethics approval was obtained from the Ethics Board of the Christian Health Association of Ghana (Ref: CHAGIRB07022021); and the National Catholic Health Service, St. John of God Hospital (ref: SJOGH/AFSR/19). The study objectives, data collection methods, confidentiality, voluntary participation and right to withdraw from the study were explained, and informed written consent was given by each of the primary caregivers of participating children prior to data collection.

## Results

Between October 2018 and February 2020, 455 children with CP were registered from the four health care facilities. The mean (standard deviation [s.d.]) age at assessment was 5.9 (4.1) years, and 42.1% (*n* = 191 out of 454) were female.

### Sociodemographic characteristics

Table 1 shows the socio-demographic characteristics of participating children. Half of the children were aged < 5 years. Overall, 31% (*n* = 138 out of 440) of the mothers and 20% (*n* = 85 out of 433) of the fathers of registered children did not receive any formal schooling. Furthermore, 18.0% (*n* = 79 out of 440) of the mothers and 28% (*n* = 124 out of 433) of the fathers had completed more than secondary education. In terms of occupation, 40.0% (*n* = 182 out of 454) of the mothers were involved in sales and service, and 31.0% (*n* = 140 out of 446) of the fathers were involved in skilled manual work. Only 12.0% (*n* = 53 out of 454) of the mothers and 2.0% (*n* = 8 out of 446) of the fathers were unemployed or not involved in any work at the time of child’s birth. Most of the households had access to an improved source of drinking water, and improved sanitation facilities. The median monthly family income was 350 [150, 725] Ghanaian Cedi (~ 29 [13–60] USD).

### Aetiology and commonly known risk factors

Table 2 summarises the aetiology of CP among children registered into the GCPR. Most children had an institutional birth. Two-thirds (65.0%, *n* = 293 out of 450) of the primary caregivers reported having complications during the birth of their child with CP, and of them, 62.0% (*n* = 182 out of 293) reported having obstructed or prolonged labour. Of the known birthweights, 21.0% (*n* = 45 out of 213) children had low birthweight (LBW). Among all children, nearly half (41%, *n* = 184 out of 452) had reported signs suggestive of birth asphyxia.

**TABLE 2:** Commonly known risk factors of cerebral palsy of children registered into the Ghana CP Register (*N* = 455).

Risk factors-subgroups	All children with CP	
	*n*	%
**Gestational age (*N* = 448)†**		
Pre-term (< 37 weeks)	233	52.0
Term (37 week or above)	215	48.0
**Birthplace (*N* = 452)†**		
Home birth	21	5.0
Institutional birth	431	95.0
**Mode of delivery (*N* = 451)†**		
Spontaneous vaginal delivery	351	78.0
Instrumental delivery	8	2.0
Caesarean section	92	20.0
**Complications during birth (*N* = 450)†**		
No	157	35.0
Yes	293	65.0
Obstructed or prolonged labour	182	62.0
Malpresentation	25	8.0
Haemorrhage	12	4.0
Pre-eclampsia/Eclampsia	16	5.0
Premature rupture of membrane	3	1.0
Multiple complications	10	3.0
Other complications	3	1.0
**Birthweight among known (*N* = 213)†**		
LBW	45	21.0
Normal BW	168	79.0
**Plurality (*N* = 449)†**		
Singleton	438	98.0
Twins	11	2.0
**Sign of birth asphyxia (*N* = 452)†**		
No	204	45.0
Yes	184	41.0
Don’t know	64	14.0
**Consanguinity (*N* = 427)†**		
No	417	98.0
Yes	10	2.0

CP, cerebral palsy.

† , Total available responses for the specific risk factors-subgroup.

### Age of diagnosis, timing of brain injury and probable cause of cerebral palsy

The mean (s.d.) age of confirmed diagnosis of CP was 22.2 (21.6) months among the registered children. More than two-thirds (70.0%, *n* = 320 out of 455) had received their diagnosis at or before 24 months of age, and only 5.0% (*n* = 24 out of 455) children received a confirmed diagnosis after 5 years of age.

Overall, 80% (*n* = 362 out of 455) of children had pre- or perinatally acquired CP, and the remaining 20.0% (*n* = 93 out of 455) had post-neonatally acquired CP. The causes of post-neonatally acquired CP included accidental head injury (58.0%, *n* = 49 out of 85), post-seizure (29.0%, *n* = 25 out of 85), unspecified infections (6.0%, *n* = 5 out of 85), stroke/cerebrovascular accident (CVA) (4.0%, *n* = 3 out of 85), post immunisation (2.0%, *n* = 2 out of 85) and viral infection (1.0%, *n* = 1 out of 85). The causes remained unknown for *n* = 8 children.

### Motor characteristics and the presence of associated impairments

Two-thirds of the children had spastic CP; of them, 56.0% (*n* = 177 out of 317) had tri- or quadriplegia. A further 11.0% (*n* = 52 out of 455) had ataxia. Calculating the aggregate total of GMFCS levels yields 61.0% (*n* = 273 out of 450) of children in this classification system.

Among all children, 70.0% (*n* = 320 out of 455) had at least one associated impairment (visual: 6.0%, *n* = 29 out of 455; hearing: 22.0%, *n* = 100 out of 453; speech: 72.0%, *n* = 328 out of 453; intellectual: 41.0%, *n* = 100 out of 247 and epilepsy: 12.0%, *n* = 49 out of 454) (Table 3).

**TABLE 3:** Motor characteristics and associated impairments of children registered into the Ghana CP Register.

Motor characteristics-subgroups	*N* = 455	
	*n*	%
**Predominant motor type (*N* = 455)†**		
Spastic	317	70.0
Dyskinesia	38	8.0
Ataxia	52	11.0
Hypotonia	48	10.0
**Topography of spasticity (*N* = 317)†**		
Mono/Hemiplegia	60	19.0
Diplegia	80	25.0
Tri- or quadriplegia	177	56.0
**GMFCS level (*N* = 450)†**		
I	59	13.0
II	119	26.0
III	44	10.0
IV	154	34.0
V	75	17.0
**Number of associated impairments (*N* = 455)†**		
None identified	135	30.0
1–2 impairments	253	56.0
Multiple impairments	67	15.0
**Visual impairment (*N* = 455)†**		
No	426	94.0
Some impairment	22	5.0
Functionally blind	6	1.0
Unknown	1	0.2
**Hearing impairment (*N* = 453)†**		
No impairment	353	78.0
Some impairment	85	19.0
Bilateral deafness	4	1.0
Unknown	11	2.0
**Speech impairment (*N* = 453)†**		
No impairment	125	28.0
Some impairment	120	26.0
Non-verbal	179	39.0
Unknown	29	6.0
**Intellectual impairment (*N* = 269)** ‡		
No	147	55.0
Mild	58	22.0
Moderate	32	12.0
Severe	10	4.0
Unknown	22	8.0
**Epilepsy (*N* = 454)†**		
No	319	70.0
Yes	54	12.0
Resolved by age 5 years	64	14.0
Unknown	17	4.0

GMFCS, gross motor function classification system.

† , Total available responses for the specific motor characteristics-subgroup;

‡ , Confirmed diagnosis could not be made for *n* = 186 children.

### Rehabilitation and educational status

Overall, 21.0% (*n* = 93 out of 454) of the registered children had never received any rehabilitation services. The mean (s.d.) age of starting rehabilitation was 25.0 (21.0) months. The major causes of never receiving any rehabilitation included parental unawareness about the need for and availability of services (62.0%, *n* = 56 out of 90) and financial constraints (32.0%, *n* = 29 out of 90) (Table 4).

**TABLE 4:** Rehabilitation and educational status of children registered into the Ghana CP Register.

Rehabilitation and educational status	*n*	%
**Rehabilitation status (*N* = 454)†**		
Received rehabilitation services	361	80.0
Never received rehabilitation services	93	20.0
**Reason for never receiving rehabilitation (*N* = 90)†**		
Unawareness	56	62.0
Financial constraint	29	32.0
Transport problem	2	2.0
Others	3	3.0
**Enrolment to school (*N* = 184)** ‡		
None	128	70.0
Mainstream	26	14.0
Special education	27	15.0
Both	3	2.0
**Reason for not enrolling the child in any education programme (*N* = 106)†**		
Lack of disability-inclusive school/education	38	36.0
Financial constraint	18	17.0
Parents’ refusal	48	45.0
Others	2	2.0

† , Total available responses for the specific risk factors-subgroup.;

‡ , Only reported for children aged ≥ 6 years at the time of assessment.

Overall, 70.0% (*n* = 128 out of 184) of school-aged children were not enrolled either in mainstream or special education schools. The leading causes for non-enrolment in any educational institution were parents’ refusal to enrol and the lack of disability-inclusive schools in the neighbourhood (Table 4).

## Discussion

To the best of our knowledge, ours is the first study reporting the epidemiology of CP among children from an institution-based register in Ghana. Most of the children in our cohort were aged below 10 years and had educated parents with varied occupations or income generating activities. Preterm birth, birth complications and birth asphyxia were frequently reported in our cohort. Most children had severe gross motor function limitations and had multiple associated impairments. In the absence of early intervention and rehabilitation services, these children are at high risk of adverse health and functional outcomes affecting their quality of life and survival in the long run (Jahan et al. 2019; Karim et al. 2019a; Power et al. 2019).

Most children registered in the GCPR had CP acquired pre- and perinatally with a high burden of commonly known risk factors recognised globally (Abubakari, Taabia & Ali 2019; Adu-Bonsaffoh et al. 2019; Jahan et al. 2020; Khandaker et al. 2019; McIntyre et al. 2013; Monokwane et al. 2017). Timely intervention is crucial to manage these risk factors and prevent adverse outcomes in LMICs. A recent study reported 80% of the health facilities in Ghana lacked sufficient capacity for basic or comprehensive emergency obstetric care for mothers (Kyei-Onanjiri et al. 2018). It is therefore important to emphasise the critical need for capacity development within the health system, as well as the need to implement preventive strategies and programmes for clinical management of children with CP in Ghana.

Nevertheless, the proportion of CP acquired post-neonatally was also high when compared with data collected from other institution-based settings (Karim et al. 2022). Furthermore, the high percentage of post-neonatally acquired CP following a head injury and post-seizures is concerning. The post-seizure complications could be related to viral or bacterial central nervous system (CNS) infections, that is meningitis and encephalitis (Germany et al. 2013), cerebral malaria or could be because of bilirubin encephalopathy (Jahan et al. 2019). Unfortunately, these data were not available but warrant further investigation. Data from meningitis surveillance between 2009 and 2013 in the upper west region of Ghana showed that 19.0% of the documented meningitis cases were among children aged < 5 years (total 46.0% aged < 14 years), and the bacterial pathogen was isolated in one-third of the cases (The RTS,S Epidemiology EPI-MAL-002 Study Group 2021). There is opportunity to mitigate risk by raising awareness of vaccine-preventable infectious disease, and health promotion (Renner et al. 2019).

Most children in the GCPR had spastic tri- or quadriplegia, GMFCS level III-V and associated impairments. This overrepresentation of severe forms of CP could be because of recruitment bias associated with the surveillance mechanism (i.e. institution-based). Community-based surveillance is required for better understanding of the clinical profile of CP among children in Ghana.

We observed that 20% of registered children had never received any rehabilitation services. This higher percentage of rehabilitation uptake among registered children could be because we recruited study participants from healthcare facilities that also provide rehabilitation services to children. Nevertheless, the reasons for non-receipt of rehabilitation services in the cohort need further exploration as the scenario may not be similar in rural and remote communities. As mentioned earlier, only a third of the population in Ghana is supported by the government-funded health insurance scheme (UoG 2018). Therefore, children from low and middle-income families who are not covered by any insurance are less likely to be able to afford out-of-pocket expenses, thus limiting their opportunity for service utilisation in an LMIC like Ghana (Al Imam et al. 2021, 2022; Jahan et al. 2021). This is a concern as studies in other LMICs have found a strong relationship between such severe forms of CP and subluxation of hips, low social participation and quality of life if not timely intervened (Karim et al. 2019b; Power et al. 2019). Early intervention and rehabilitation service providers should consider working towards reducing the inequity in service provision for children with disability (e.g. CP) in LMICs like Ghana.

Most school-aged children in GCPR were not enrolled in a mainstream or special education system, with parental refusal being the prime reason. This is possibly because of the stigma and social exclusion imposed by family, community, society and culture in LMICs like Ghana (Nketsia et al. 2019; Patel et al. 2017; Zuurmond et al. 2022). Additionally, a lack of disability-inclusive schools as well as physical barriers to mainstream schools can reduce opportunities to access education for vulnerable children. A similar situation was reported in a recent qualitative study in Ghana, where the mothers of children with CP shared their experience of their children being excluded from mainstream or special education system because of a lack of facilities (Nketsia et al. 2019). Interventions and programmes to promote inclusive education and training of educators and schoolteachers could be a start to overcome the barriers. More in-depth information is required to understand parents’ perspective for refusal to enrol their children for formal schooling and promote an evidence-based approach to improving the situation (Smythe, Adelson & Polack 2020; Zuurmond et al. 2019).

Despite considerable efforts, our study had several limitations. Firstly, we used an institution-based surveillance mechanism, and the participants were recruited from four tertiary hospitals which also are the highest referral centres for the region. As the data in this study only represent children accessing these services, the findings may not be generalisable to the population level. Nevertheless, data reported in this study are the first of its kind and have provided an understanding of the epidemiology of CP among children in Ghana. These data could also be used as an infrastructure to scale up the CP register activities at the population level. Secondly, the aforementioned recruitment bias may indicate that the rehabilitation status of the registered children may vary from the true scenario. Population-based surveillance could overcome this limitation. Thirdly, a large proportion of the children in GCPR were of young age (< 5 years); they need to be followed-up and reassessed after their fifth birthday to document their CP descriptions. However, as most children in the cohort had spastic, dyskinetic or ataxic CP, their diagnosis is likely to remain same at follow-up assessments, although their motor function level may vary. Fourthly, this study solely focused on describing the epidemiology of CP among children in Ghana, without assessing associations between various variables. Although the findings align with the study’s objective, further analysis exploring the relationships between socio-demographic factors, aetiology, clinical and motor types, and rehabilitation status would be valuable for developing targeted interventions for children with CP in Ghana. Fifthly, the data for this study were collected between October 2018 and February 2020. While the data are somewhat dated, the findings remain significant as this is, to our knowledge, the first detailed epidemiological study of CP among children in the country. Finally, there were a large number of missing data for the following variables: monthly family income (*n* = 118 respondents did not want to report their income), birthweight (was unknown for *n* = 242 children), presence of intellectual impairment (diagnosis could not be confirmed for *n* = 186 children) and reasons for non-school-attendance (*n* = 22 did not mention any causes). As mentioned in the statistical analysis section, we therefore only used descriptive statistics and reported valid percentages throughout the results section.

In the past decade, the understanding of epidemiology of CP in different LMICs has improved. The growing evidence has also highlighted regional differences and the need for country-specific data to better understand the epidemiology of CP in low-resource settings. Our study has added important data to advance the global knowledge base of CP in LMICs. Data from the GCPR could act as a baseline, provide an institutional framework for future translational research and contribute to the development of strategies and intervention for prevention, clinical management, rehabilitation and education of children with CP in Ghana.
